# Unusual presentation of moyamoya disease with popliteal involvement: case report and review of the literature

**DOI:** 10.1590/1677-5449.200216

**Published:** 2021-06-21

**Authors:** Mustafa Etli, Oguz Karahan

**Affiliations:** 1 Alaaddin Keykubat University, Medical School, Alanya/Antalya, Turkey.

**Keywords:** moyamoya disease, cerebrovascular disease, popliteal artery, unusual involvement, doença de moyamoya, doença cerebrovascular, artéria poplítea, envolvimento incomum

## Abstract

Moyamoya disease is a rare disorder that involves the cerebrovascular system. Usually, it leads to occlusion of the arteries of the cerebral system and causes cerebral circulatory complaints. A 48-year-old female patient was admitted to our clinic with intermittent claudication in both legs. Biphasic and monophasic waveform patterns were detected bilaterally in distal (trifurcation arteries) lower extremities with Doppler sonography. The patient therefore underwent systemic vascular examination. Computed tomography angiography revealed bilateral carotid occlusion at the level of supraclinoid segments, and opacifications were detected at the distal segments of the bilateral anterior cerebellar and middle cerebellar arteries. The patient was diagnosed with moyamoya disease, and anticoagulant treatment was started. In conclusion, most previous reports have presented the cerebrovascular involvement of moyamoya disease. However, this disease can involve different peripheral vascular systems and careful and systemic vascular examination is necessary for an exact diagnosis.

## Resumo

A doença de moyamoya é um distúrbio raro que envolve o sistema cerebrovascular. Normalmente, leva à oclusão das artérias do sistema cerebral e causa problemas circulatórios no cérebro. Uma mulher de 48 anos foi admitida em nossa clínica com claudicação intermitente em ambas as pernas. Na ultrassonografia com Doppler, foram detectados padrões de formato de onda bifásico e monofásico nas extremidades inferiores distais (artérias da trifurcação) de forma bilateral. Portanto, realizou-se o exame vascular sistêmico na paciente. A angiografia por tomografia computadorizada revelou oclusão carotídea bilateral no nível dos segmentos supraclinoides, e opacificações foram detectadas nos segmentos distais das artérias cerebelares anteriores e médias de forma bilateral. A paciente foi diagnosticada com doença de moyamoya, e o tratamento anticoagulante foi iniciado. Em conclusão, a maioria dos relatos anteriores apresentou o envolvimento cerebrovascular da doença de moyamoya. No entanto, essa doença pode envolver diferentes sistemas vasculares periféricos, e um exame vascular sistêmico minucioso é necessário para um diagnóstico exato.

## INTRODUCTION

Moyamoya disease (MMD) is a chronic progressive cerebrovascular disease that leads to spontaneous occlusion of the main branches of internal carotid arteries (ICAs) and loop of Willis. This occlusion leads to growth of fine collaterals in the cerebrovascular system to compensate and supply blood flow requirements.^1^^,^^2^ According to previous reports, the disease is more frequent in females, and the incidence of reported cases is higher in the Japanese population.^3^ The disorder is associated with fatal outcomes because of the fine collaterals that may form arterial aneurysms and provoke bleeding due to their fragility. The most common complaints are usually related to the cerebrovascular system and are caused by reduced blood flow and intracerebral bleeding.^4^

Here, we report an adult moyamoya case that presented with bilateral popliteal artery involvement.

## CASE REPORT

**Figure 1:**
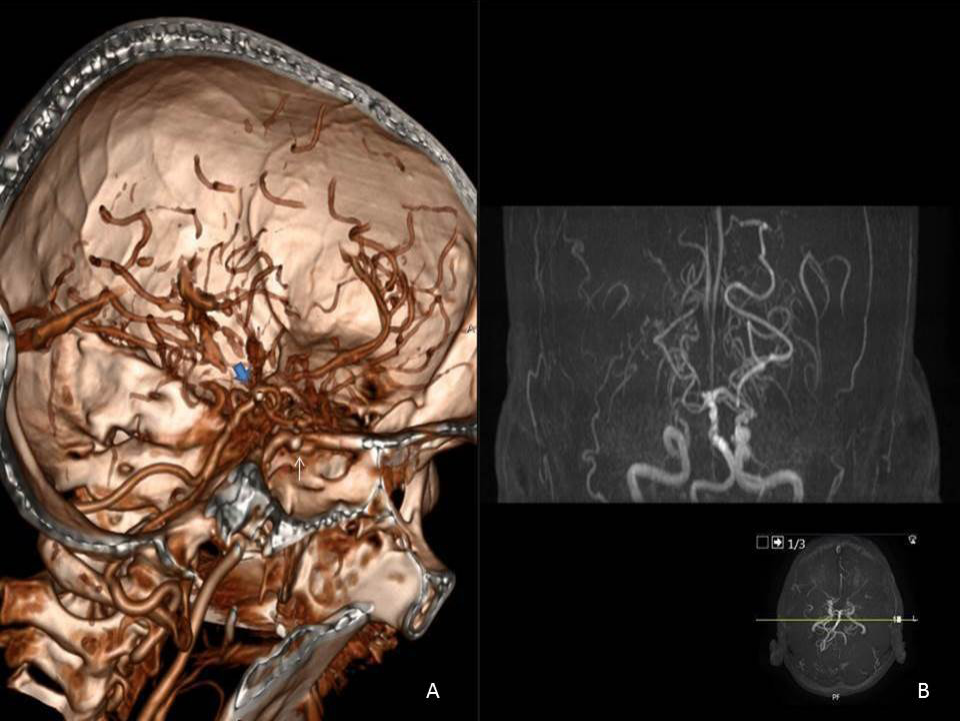
(A) Contrasted computed tomography scans of cranium revealing internal carotid artery occlusion at the level of supraclinoid segments (white arrow) and opacifications of distal segments of bilateral anterior cerebellar and middle cerebellar arteries (blue arrow); (B). Abnormal coronary collaterals (puff of smoke sign).

**Figure 2:**
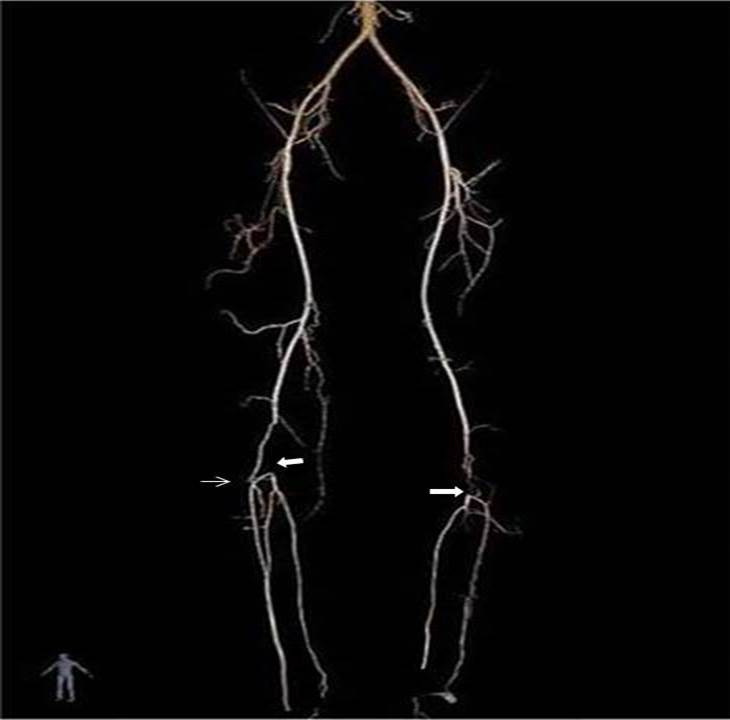
Computed tomography angiography of lower limbs: Bilateral popliteal occlusions (white arrows) and opacifications can be seen.

A 48-year-old woman was admitted to hospital due to claudication at 250 to 300 meters and tremor in both legs. During history-taking, she reported periodic headaches and vertigo complaints lasting years, she described that she had tremors and muscle weakness during her childhood, she had had one lost pregnancy, and her mother does not have diabetes. She has no history of smoking. Family history for cardiovascular disorders was negative and she explained that her mother’s family has a history of diabetes mellitus. She indicated that her father was hypertensive. However, the patient's own blood pressure was normotensive (120/70 mmHg). Assessment of the patient’s physical and ethnic characteristics determined that she was of Turkish origin (European), 52 kg in weight, and 1.63 m in height. Ankle brachial indexes were calculated as 0.72 on the right side and 0.78 on the left side. Biphasic and monophasic waveform patterns were detected bilaterally in distal (trifurcation arteries) lower extremities with Doppler sonography. Complete blood count and biochemical test results were studied to evaluate systemic risk factors. However, whole blood test results were within normal ranges (Table 1). Thereafter, computed tomography angiography (CTA) was planned for the lower extremities, as well as for the cerebrovascular system because of tremor and cerebrovascular complaints. Cerebrovascular CTA revealed bilateral carotid occlusion at the level of supraclinoid segments, and opacifications were detected at the distal segments of bilateral anterior cerebellar and middle cerebellar arteries (Figure 1A). Additionally, abundant collateral formations (puff of smoke sign) were detected in the cerebral parenchyma and basal ganglia (Figure 1B). Lower extremity CTA revealed bilateral segmental occlusions of both popliteal arteries (Figure 2). The patient was diagnosed with moyamoya disease, and anticoagulant and antiaggregant treatment was started at a dose of 2×1 6000 IU enoxaparin sodium for the first ten days and 300 mg/daily acetylsalicylic acid. Additionally, subsequent 60 mg daily prednisolone treatment was started because of insufficient exclusion of vasculitis. There are no strict guidelines on when to obtain ethical clearance for writing and publishing a case report. We therefore obtained signed informed consent from the patient.

**Table 1:** Routine laboratory test results for the patient.

Parameters	Value	Ranges
Platelets 10^^3^/µL	397	100-300
Red Blood Cell Distribution 10^^6^/µL	4.13	4.0-5.2
White Blood Cell Distribution 10^^3^/µL	9.39	4.5-11.0
Hemoglobin g/dL	12.73	12.0-16.0
Blood Urea Nitrogen mg/dl	19	15-43
Creatinine mg/dl	0.69	0.6-11
High Density Lipids mg/dl	50.9	25-65
Cholesterol mg/dl	198	119-199
Triglycerides mg/dl	144	39-149
Low Density Lipids mg/dl	118.3	100-150
Albumin g/dl	4.2	3.5-5.0
Glycated hemoglobin (HbA1c) [%]	5.11	4-6.4
Fasting Glucose	89	70-109

## DISCUSSION

Takeuchi and Shimizu described a case of an unknown disease characterized by bilateral dysplasia of internal carotid arteries.^5^ The etiopathogenesis of the disease is still unclear. However, hereditary transition was described in a small part of the Japanese population.^1^ Increased proangiogenic factors and growth factors were detected in samples of cerebrospinal fluid, the temporal artery, and the dura.^6^^,^^7^ The disease has two peaks in life at 5 years and at about 40 years.^8^ There was no family history in the case presented.

Previously reported cases were commonly admitted to hospital with cerebral findings due to ischemia and hemorrhage.^9^ Ischemic components were related to occlusion of main arteries such as internal carotid arteries (ICAs) and loop of Willis. The common pathology is described as proliferation of smooth muscle cells and intimal thickening that leads to luminal stenosis in the ICA and loop of Willis. Hemorrhage is related to fine fragile collaterals and aneurysmatic formations.^9^ The diagnostic criteria were modified in 2015 as “certain bilateral involvement is unnecessary” from the first criteria determined in 1997, as follows:

- ICA occlusion/stenosis at the terminal segment and/or anterior and/or middle cerebral arteries at the proximal segment;
- Abnormal collateral vessel network (puff of smoke appearance);
- Bilateral involvement.^1^^,^^9^

For diagnostic assessment, cerebral angiography, magnetic resonance imaging and angiography, and positron emission tomography are often recommended.^1^^,^^9^ However, computed tomography angiography (CTA) or direct computed tomography scans were used for monitoring some series.^10^ We used peripheral Doppler ultrasound for initial diagnosis as the primary complaint of our patient was claudication. Subsequently, CTA was used to scan both peripheral and cerebral circulation.

An angiographic staging system was described by Mugikura et al.^11^ The Mugikura et al.^11^ stages are presented in Table 2.^12^ According to this system, the patient described seems to be compatible with the second stage. However, this staging system only includes the cerebrovascular system. The difference in the patient described is that she presented with lower limb vascular involvement.

**Table 2:** Angiographic staging according to Mugikura et al.^11^

Occlusion of ICA Stage	Angiographic Findings
I	Mild to moderate stenosis around carotid bifurcation with absent or slightly developed ICA moyamoya
II	Severe stenosis around carotid bifurcation or occlusion of either of proximal ACA or MCA with well-developed ICA moyamoya
III	Occlusion of both proximal ACA and MCA with well-developed ICA moyamoya
IV	Complete occlusion of both proximal ACA and MCA with absent or small amount of ICA moyamoya

Weber et al.^12^ described the first case of upper and lower limp involvement in an adult woman with typical moyamoya findings. They found diffuse luminal stenosis and concentric thickening of both common and superficial femoral artery walls.^12^ Differently, Kaczorowska et al.^13^ detected stenoses of the aorta, celiac trunk, and bilateral renal arteries. Ramesh et al.^14^ described external iliac stenosis in a 14-month-old girl. To the best of our knowledge, we present the first case of classical moyamoya disease that is accompanied by popliteal artery involvement without proximal involvement of the lower limb arteries.

Commonly, two medical treatments are recommended for moyamoya disease: antiaggregant and anticoagulant treatment.^1^ Therefore, we started these regimens as described in previous reports. Moreover, anticoagulation is a risk for cerebral hemorrhage in adult patients. However, anticoagulation is recommended in the acute phase in normotensive patients for cerebrovascular protection.^15^ We started with enoxaparin sodium for first ten day and 300 mg/daily acetylsalicylic acid. We could not exclude vasculitis due to the insufficient laboratory infrastructure. Weber et al.^12^ prescribed steroid therapy for insufficient exclusion of vasculitis. Similarly, we started steroid treatment for the same reason.

In conclusion, peripheral involvement of moyamoya disease is not described comprehensively in previous reports. However, as shown in our case, this disorder can involve limb arteries. Additionally, claudication can be the main symptom. Therefore, patients with peripheral occlusion complaints should be examined for systemic findings that could be related to ischemia involving other organs. Moreover, we suggest that the systemic involvement of moyamoya disease should be remembered to understand the nature of the disease clearly.

### Study limitations

The main limitation of this study is the lack of genetic testing of the patient. Another point is that a histopathological diagnosis could not be made due to lack of vascular biopsy.
